# Bloody semen, severe hypertension and a worried man

**DOI:** 10.11604/pamj.2015.20.326.6194

**Published:** 2015-04-06

**Authors:** Tamaraemumoemi Emmanuella Ambakederemo, Sotonye Tamunobelema Dodiyi-Manuel, Ikenna Desmond Ebuenyi

**Affiliations:** 1 Department of Medicine, Niger Delta University, Nigeria; 2 Department of Medicine, University of Port Harcourt Teaching Hospital; 3 Department of Medicine, Niger Delta University Teaching Hospital, Okolobiri, Nigeria

**Keywords:** Haematopermia, severe uncontrolled hypertension, worried man

## Abstract

Haematospermia is often associated with severe uncontrolled hypertension. The bloody semen is often very worrisome for the patient and his sexual partner(s). In addition to anti-hypertensive, counselling and lifestyle modification are essential for management of the condition.

## Introduction

Haematospermia is defined as blood in the semen. It is usually a benign and self-limiting condition. It is often idiopathic and severe uncontrolled hypertension has been implicated in some cases [1]. The psychological impact of the condition may be of more consequence than the physical impact as the case below illustrates.

## Patient and observation

We report a case of 38-year-old businessman who presented to the medical outpatient clinic of the Niger Delta Teaching hospital here in Nigeria, with a 2-week history of bloody ejaculate. He had 3 episodes with bright red blood noticed in the ejaculate. There was no associated history of urgency, urge incontinence or of dysuria. He was diagnosed hypertensive 5 months prior to onset of symptom but had not been regular with oral anti-hypertensives and follow-up clinic visits. There was no history of trauma to the genitals or prostate. No history of scrotal pain, no bleeding from any other part of his body, no weight loss, no chronic cough, no noticeable fever or drenching night sweats. He was not a known diabetic or sickle cell disease patient. He had been taking oral sildenafil intermittently for 2 years to enhance sexual performance. The patient had a positive history of unprotected sexual intercourse with current and previous girlfriends. His father and elder sister were known hypertensives. He drank alcoholic beverages occasionally but was a lifelong non-smoker. On examination, he was not in any obvious respiratory or painful distress, not pale, anicteric, afebrile to touch, acyanosed, no digital clubbing, no significant peripheral lymph node enlargement, and no dependent oedema. There were no signs of Cushing′s syndrome, acromegaly, or systemic sclerosis. He was conscious and alert, well oriented in time, place and person, scoring 10/10 on the abbreviated mental test score. He had no obvious focal neurologic deficits. His arterial walls were thickened and locomotor brachialis was present. Radiofemoral delay and renal bruits were absent. His blood pressure was 200/120 mmHg. The apex beat was at the 6th left intercostal space, lateral to the mid-clavicular line, heaving. The first, second and fourth heart sounds were present, all of normal intensity with no murmurs heard. The respiratory rate was 20 cycles per minute. Respiratory system examination revealed no abnormalities. Abdominal examination was normal. Genito-urinary system examination including prostate examination was also normal. A diagnosis of severe hypertension and haematospermia was made.

Investigation results included a full blood count that revealed a haematocrit of 34% while other parameters were within the normal range. Serum chemistry showed increased urea levels (urea 11.5 mmol/l), and elevated creatinine (300/mol/l). His lipid profile revealed mildly elevated LDL cholesterol (3.4 mmol/l) whiles other lipid parameters were normal. His fasting blood sugar was normal. Immunological testing showed no evidenced of HIV or tuberculosis. Radiography of the chest showed cardiomegaly with left ventricular preponderance but no evidence of aortic coarctation. Electrocardiography showed sinus rhythm with increased P-terminal force (left atrial enlargement), T-wave inversion in leads II, III, aVF, V5 and V6, left ventricular hypertrophy (Sokolow Lyon voltage criteria SV1 + RV5 = 41 mm). Dipstick urinalysis showed trace glycosuria but no protein or blood. Urine microscopy revealed 0 - 3 pus cells per high power field but showed no casts. There was no significant bacterium growth on urine culture. Seminal fluid analysis revealed a brownish gelatinous fluid with a volume of 2.5 mL, pH 8.5 (7.2 - 7.8), numerous red blood cells, 0 - 5 pus cells per high power field, the spermatozoa count 10.2 x 10^6^ ml with 30% being actively motile, sluggishly motile 10%, non-motile 60%, normal morphology 60%, abnormal morphology 40%. Seminal fluid culture did not have any significant yields. Fundoscopy was not done. Renal ultrasound scan, echocardiogram, prostate scan and prostate specific antigen were also ordered for but were not done on account of financial constraints. The patient was admitted and counseled on his clinical condition, lifestyle modification and DASH (dietary approach to stop hypertension) diet. His blood pressure was lowered gradually, over several days, with oral nifedipine control release tabs, tabs moduretic, and lisinopril and he discharged home after 2 weeks at which time his blood pressure was 150/100 mm Hg. An appointment for follow-up visit was made (cardiology and urology) for the following week. In follow-up visits, he complained that when his blood pressure was measured outside the hospital, it was significantly less than the values obtained at the hospital. He also had the problem of his sexual partners running scared and avoiding him once they discover the haematospermia. Some weeks after being on regular anti-hypertensives, the haematospermia resolved completely. He was however lost to follow-up, as his family members convinced him that his problems had been a “spiritual attack” and he resolved to seek further treatment at a church. About three months after last visit, he is said to have suffered a stroke while still at the church (this information was gotten from one of his relations as we made attempts to contact him). He is yet to be seen or heard from since.

## Discussion

The true prevalence of haematospermia is unknown. It is likely that many cases escape the patient′s notice and remain unrecognized and under-reported. Haematospermia is most commonly caused by infection or injury-including iatrogenic injuries such as transrectal prostate biopsy, urethral instrumentation, haemorrhoid sclerosing injection etc [2]. Other conditions of the prostate include prostate cancer (2% of cases), prostatitis, prostate telengiectasia, varices, calculi, brachytherapy for prostate cancer. Urethral causes include urethritis, urethral cysts, polyps, condylomata, strictures. Seminal vesicle lesions include congenital or acquired cysts, amyloidosis. Infections include tuberculosis, human immunodeficiency virus disease, cytomegalovirus infection, herpes simplex virus, Chlamydia trachomatis, enterococcus faecalis, ureaplasma urealyticum, schistosomiasis, hydatid disease. Other causes are testicular and perineal blunt trauma, urethral self instrumentation, systemic diseases such as hypertension, chronic liver disease, amyloidosis, lymphomas, bleeding diathesis e.g Von Willebrand disease. Severe hypertension causes an estimated 5% of cases of haematospermia [3]. The exact mechanism by which it does this is unclear but may have similar basis to association of hypertension with epistaxis. Severe hypertension according to the WHO definition is a blood pressure ≥180/110mmHg [4].

The patient in this case report has a presenting blood pressure of 220/110mmHg. Risk factors for haematospermia in hypertension include; severe uncontrolled hypertension, increased serum creatinine levels, severe proteinuria, renovascular disease [5]. Kurker et al identified hyperuricaemia as a possible cause of haematospermia [6]. Close et al [7] in their study suggested that the association of haematospermia is with severe uncontrolled hypertension as opposed to hypertension per se. They based this hypothesis on comparison of a small series of patients who had both haematospermia and hypertension with matched control patients with just hypertension. The former group were found to have significantly higher systolic and diastolic blood pressures, higher serum creatinine, and higher left ventricular voltages based on ECG analysis [7]. Apart from haematospermia, our patient was asymptomatic-as are around 10% of people with malignant hypertension [8]. Nonetheless, left- ventricular hypertrophy, glycosuria and deranged blood chemistry indicated end-organ damage, consistent with severe, longstanding hypertension. This finding is in line with the hypothesis propounded by Close et al [7]. It is also similar to a case report finding by Fleming et al [9]. Our patient was placed on the DASH diet in addition to anti-hypertensive medications and made good improvements in his blood pressure control within a short time. The DASH diet is a flexible and balanced eating plan that has been shown to be effective in lowering blood pressure in as little as two weeks [10]. The diet is such that it is low in saturated fat, cholesterol and total fat; focuses on vegetables, fruits and fat-free or low fat dairy products as well as whole grain.

## Conclusion

Patients presenting with haematospermia are often worried about their peculiar presentation and often need reassurance in addition to optimal blood pressure control. In addition, we emphasize the need for blood pressure monitoring in patients presenting with haematospermia and the use of a DASH diet in addition to anti-hypertensives.
